# Imaging the neural correlates of neuropathic pain and pleasurable relief associated with inherited erythromelalgia in a single subject with quantitative arterial spin labelling

**DOI:** 10.1016/j.pain.2011.12.012

**Published:** 2012-05

**Authors:** Andrew R. Segerdahl, Jingyi Xie, Kathryn Paterson, Juan D. Ramirez, Irene Tracey, David L.H. Bennett

**Affiliations:** a Nuffield Division of Anaesthetics, Nuffield Department of Clinical Neurosciences, Centre for Functional Magnetic Resonance Imaging of the Brain (FMRIB), University of Oxford, Oxford, Oxfordshire, UK; b Wolfson Centre for Age-Related Disease, Kings College London, Hodgkin Building, Guys Campus, SE1 1UL London, UK

**Keywords:** Neuropathic pain, Erythromelalgia, Arterial spin labelling, FMRI

## Abstract

We identified a patient with severe inherited erythromelalgia secondary to an L858F mutation in the voltage-gated sodium channel Na_v_1.7. The patient reported severe ongoing foot pain, which was exquisitely sensitive to limb cooling. We confirmed this heat hypersensitivity using quantitative sensory testing. Additionally, we employed a novel perfusion imaging technique in a simple block design to assess her baseline erythromelalgia pain vs cooling relief. Robust activations of key pain, pain-affect, and reward-related centres were observed. This combined approach allowed us to confirm the presence of a temperature-sensitive channelopathy of peripheral neurons and to investigate the neural correlates of tonic neuropathic pain and relief in a single subject.

## Summary

Using a composite diagnostic-neuroimaging approach, this study shows the modulatory effects of pleasant cooling on pain-related cortical regions in a subject suffering from inherited erythromelalgia.

## 1 Introduction

Erythromelalgia is characterised by severe burning pain associated with reddening of the extremities [27,37]. Inherited erythromelalgia (IEM) has been shown to be due to gain-of-function mutations in the SCN9A gene encoding the voltage-gated sodium channel Na_v_1.7 [13,16,40] that is selectively expressed in sensory and autonomic neurons [34]. Sufferers of this disease often report pain “attacks,” which are triggered by mild warming stimuli (eg, wearing socks or exercise). Many patients report that cooling of the affected limb can ameliorate pain [16,37], and this correlates with the biophysical finding that the hyperpolarizing shift in activation produced by IEM mutations can be partially normalized by cooling [17]. The strong temperature dependence of this condition provides a means to link a molecular lesion causing aberrant peripheral nociceptor function and the resulting higher cortical processing related to ongoing pain perception.

Currently, our understanding of the neural correlates of chronic, ongoing pain is minimal. A key reason for this is due to an inability to safely and noninvasively image neural activity across a whole brain during periods of tonic pain. Previously, chronic pain was investigated with the powerful but very invasive and expensive technique positron emission tomography [6,10,12,21,22,25,35]. Excitingly, an emerging body of work suggests that the noninvasive neuroimaging technique arterial spin labelling (ASL) is optimally suited to investigate these aspects of the pain experience without the need for radioactive tracers or multiple repeat scans.

ASL is a quantitative perfusion imaging technique that measures changes in neural activity based on related fluctuations in the local cerebral blood flow (CBF). Because ASL is minimally affected by low-frequency drift that undermines blood-oxygen-level-dependent (BOLD) functional magnetic resonance imaging (fMRI) in a tonic stimulus paradigm, it is possible to image changes in cortical activity that unfold over long (ie, on the minute timescale) periods of time [1,38]. To date, ASL pain studies are few. However, recent investigations by Owen [29,30] and Howard [19] provide preliminary evidence that ASL is well suited to observe changes in brain activity related to different ongoing pain states [19,29,30].

The aim of the current investigation was to interrogate a rare inherited neuropathic pain condition using a composite experimental procedure that included molecular genetics, quantitative sensory testing (QST), and ASL fMRI in a single subject. The goal of this molecule-to-man approach is to link the unique neurophysiological properties associated with a point-mutation in the sodium channel Na_V_1.7 to higher cortical pain processing.

## 2 Methods

A single erythromelalgia sufferer (female, right handed, age 32 years) participated in this study. The subject was recruited as part of the Pain in Neuropathy Study (National Research Ethics Committee UK no. 10/H07056/35) and complied with experimental studies in humans (Declaration of Helsinki). The subject gave her permission for clinical details to be written up as a case report.

### 2.1 Assessment of genotype: molecular genetic assessment

Following informed consent, all 26 coding exons of the SCN9A gene were sequenced (Department of Gastroenterology, Radboud University Nijmegen Medical Center, The Netherlands).

### 2.2 Quantitative sensory testing and experimental pain paradigm

A full quantitative sensory testing (QST) protocol was conducted in accordance with the German Research Network on Neuropathic Pain [32]. The standardised protocol measures 13 psychophysical parameters through 7 tests, which may be divided into the following groups: (1) Thermal testing for detection and painful thresholds for cold, warm, and paradoxical heat sensations; (2) Detection thresholds of touch and vibration; (3) Mechanical pain sensitivity including stimulus response functions for pinprick sensitivity, summation of pain following repetitive stimulation, and dynamic mechanical allodynia.

Results from each parameter, irrespective of units of measurement for each test, can be graphically represented for both the control and test sites as a QST profile. Profiles for each site can be compared with the bank of control data by *z*-transformation of each parameter by the equation [11]:

$$z\text{-score}=(X_{\text{single patient}})-{\text{Mean}}_{\text{controls}})/{\text{SD}}_{\text{controls}}$$

The resultant *z*-transformed profile represents all parameters as standard normal distributions where the mean equals zero. As proposed by Rolke et al. (2006), the algebraic sign of the score for each parameter was adjusted to represent the patient’s sensitivity [32]. Positive *z*-values indicate a gain of function, where the patient is hypersensitive to the test stimulus relative to controls. Conversely, negative *z*-values indicate a loss of function where the patient is less sensitive.

A 95% confidence interval (CI) of a standard normal distribution is used to define the control population, represented in Fig. 3 by the grey area. This CI is established by:

$$95\%\;\text{CI}={\text{Mean}}_{\text{controls}}\pm 1.96\;{\text{SD}}_{\text{controls}}$$

**Fig. 1:**
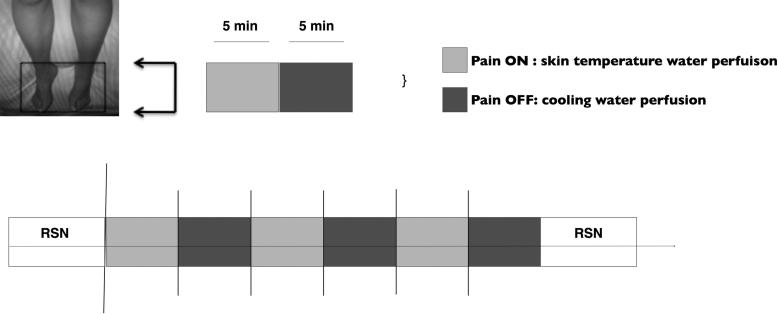
Graphic representation of the erythromelalgia psychophysical paradigm. For clarity, each coloured block represents the temperature of water continuously perfusing over the subject’s feet: “BASE” (mean temperature = 35.5°F, SE = 0.72; light gray) vs “cool” (mean temperature = 13.9, SE = 0.86; dark gray). The bottom panel shows the arterial spin labelling scan paradigm used. A 10-minute resting-state (RSN) was acquired at the beginning and end of the paradigm. Five-minute blocks of continuous erythromelalgia-associated pain (BASE, light blue) vs pain-relief (cool, dark gray) were repeated 3 times per scan session. Each scan was separated by approximately 3 minutes. Two separate scan sessions were acquired for this subject. Black bars show when the pain intensity and unpleasantness ratings were taken (verbal report, NRS).

A graphic representation of the experimental stimulus paradigm employed is displayed in Fig. 1. A motorized water perfusion system was made in-house at the Centre for Functional Magnetic Resonance Imaging of the Brain (FMRIB) by modifying parts and machinery from an “Iceman Motor Hose” kit sold by DJO UK Ltd (Guildford, Surrey, UK). Specifically, the premade kit allows for a well-maintained temperature water solution to be continuously perfused over the subject’s feet, which are wrapped in foot-shaped pads that circulate water over the skin surface. We modulated the kit by bifurcating the circulation route such that each foot was stimulated by the same water bath solution. Two separate water baths were used to generate each stimulus condition. The motorized water coolers were kept in the scan console room as they were not MR compatible. A 3-meter-long insulated perfusion tube directed the water flow from the coolers to the foot pads placed on the subject. Water was able to continuously circulate between the pads and the coolers without any obstruction to the scan session. Additionally, temperature levels of each water cooler were continuously maintained throughout the scan session using thermometers. An experimenter would place the foot-shaped circulation pads over the subject’s feet in between each scan block to effectively ramp the stimulus condition to the desired setting (ie, cooling vs erythromelalgia-associated baseline).

We did not scan these ramp sessions because we needed to speak with the subject on a frequent basis to ensure that the stimulus condition was inducing an appropriate experience and that the subject was awake and able to participate in the scan. Each ramp session took approximately 3 minutes.

Phases of erythromelalgia baseline pain (BASE) were induced with a warm solution (average temperature 35.5°C, SE 0.72) that was below the heat pain threshold defined by QST (average temperature 43°F, SE 0). Phases of pain relief (COOL) were induced with a cooling water solution (average temperature 13.9°C, SE 0.86).

Brain activity was recorded during 3 5-minute blocks of each BASE and COOL stimulus temperature using quantitative perfusion functional imaging. Each stimulus block was an independent scan. Because there was no reliable way to stimulus-lock the onset of the subject’s perceptual change with the stimulus condition and the first acquisition volume, the scan was started only after the subject verbally reported that the pain had ceased (numerical rating scale [NRS] = 0; COOL) or was strong (NRS > 5; BASE). Each scan was exactly 5 minutes long. Pain intensity ratings were monitored verbally using an NRS (0 = no pain; 10 = worst pain) at the very beginning and end of each stimulus block. Additionally, after each COOL block, a pleasantness score (NRS: 0 = not pleasant; 10 = most pleasant) was taken.

### 2.3 MR parameters

The subject was scanned with a pseudo-continuous ASL (pCASL) sequence [11] using gradient-echo echo planar imaging readout (repetition time = 3.75 seconds, echo time = 13 ms, full k-space). Twenty-four axial slices in ascending order (3 × 3 × 4.5 mm voxels, 0.5 mm inter-slice gap) were prescribed for the subject, providing whole brain coverage (including brainstem and cerebellum). The labelling plane was chosen optimally for the subject based on a time-of-flight scan of the neck, located ∼8–10 cm inferior to the centre of the 26 slices. A 90° presaturation pulse was applied before the labelling pulses. The labelling duration was 1.4 seconds and 5 different post-labelling delay (PLD) times were adopted. The 5 PLD values (0.64 seconds, 0.792 seconds, 0.896 seconds, 1 seconds, 1.152 seconds) were generated according to Optimal Sampling Schedule theory [7,39]. PLD times were pseudo-randomised per repetition time. The same order was applied to every subject; 16 PLD values were applied for each 2-minute stimulus block. The subject was scanned using a Siemens 3T Verio system (Erlangen, Germany) fitted with a 32-channel head coil.

### 2.4 Image analysis

Imaging data were analysed using the FMRIB Software Library [33]. All data were pre-processed using standard FMRIB Software Library tools in native space.

At the first level, data were preprocessed using MCFLIRT (motion correction [23]), BET (brain extraction [24]), spatially smoothed (full width at half maximum = 5 mm) and high pass filtered (320 seconds). Data were de-noised of nonphysiological artefacts through visual inspection of independent components [26]. All analysis was carried out in native space. At the first level, the general linear model was adopted for modelling CBF and any contaminating BOLD effects. At the second level, a model of each stimulus condition and relevant contrasts (eg, BASE > COOL) was fit to the subject’s perfusion time series using a fixed-effects analysis. At the top level, a fixed-effects analysis was used to observe the average CBF changes specific to each contrast (ie, BASE > COOL: group mean CBF changes showing regions more active during pain vs cooling). Cluster-based thresholding was used to generate clusters of CBF changes showing effects with a significance level of *P* < 0.05, using a *z*-score cut-off of 2.3.

## 3 Results

### 3.1 Case report

**Fig. 2:**
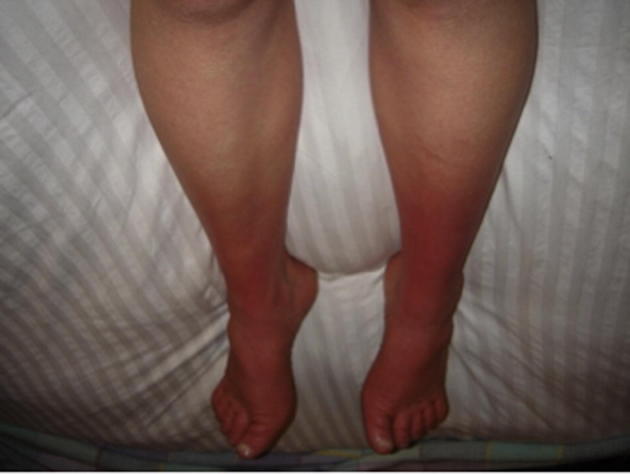
Photograph of the patient’s legs showing erythema up to the level of the mid calf.

The patient was assessed at the age of 29 years old. She presented at birth with erythema of the hands and feet and as soon as she was able to talk complained of hot painful feet. Initially she described fluctuations of her symptoms with exacerbations of pain and erythema triggered by warmth, exercise, alcohol, and wearing enclosed shoes; her symptoms could be alleviated by cooling. She had previously developed foot ulceration as a consequence of excessive cooling in cold water and at the age of 16 years received epidural anaesthesia to control her pain. She currently reports constant foot pain. Her pain has partially responded to topical 5% lidocaine plasters (applied to the feet for 12 hours a day) and she had also derived some relief from treatment with imipramine 100 mg daily. There is no family history of erythromelalgia; her parents and brother are unaffected. On examination she had erythema of the fingers and feet extending up to the mid calf (Fig. 2). Neurological examination was normal.

**Fig. 3:**
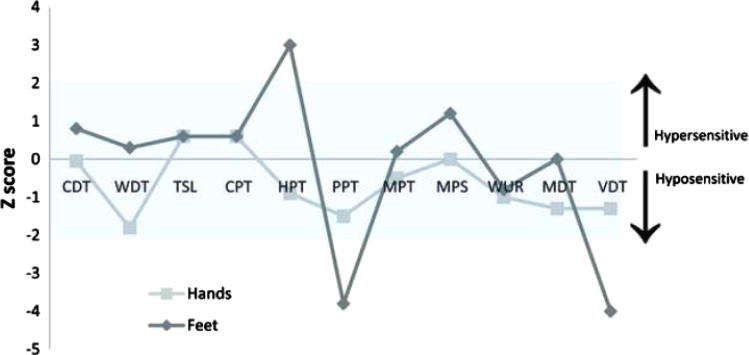
Quantitative sensory test results. Quantitative sensory tests were performed on both hands and feet. These same tests have been repeated in normal healthy controls to comprise an extensive database as generated and held by the German Neuropathic Pain Network (DFNS) (8). These normal data are distributed within the shaded area (mean at 0 ± 2 SDs). Data from our subject are reported as *z*-score profiles for each sensory test as depicted here. *z* Score is defined as the SD of the recorded result from the mean normal data result. Each data point is discrete, however, they are connected for graphical illustration as a *z* profile. Quantitative sensory tests included: CDT (cold detection threshold), WDT (warm detection threshold), TSL (thermal sensory limen), CPT (cold pain threshold), HPT (heat pain threshold), PPT (pressure pain threshold), MPT (mechanical pain threshold), MPS (mechanical pain sensitivity), WUR (wind up ratio), MDT (medical detection threshold), and VDT (vibration detection threshold). All tests to the control site were within normal limits. Hypersensitivity to heat pain is demonstrated by a lowered HPT in the feet. Hyposensitivity to deep pressure (PPT) and vibration (VDT) is also seen in the feet. These abnormal sensitivities may be attributable to thickening of the skin at the test site. Additionally, the presence of paradoxical heat sensations was tested using TSL. Three paradoxical heat sensations were recorded in the feet (an abnormally high number), whilst there were none in the hands.

A full QST protocol [32] was conducted in accordance with the German Research Network on Neuropathic Pain (Fig. 3). This demonstrated hypersensitivity to noxious thermal stimuli applied to the feet (her heat pain threshold was reduced by 3 SDs from the mean of gender- and age-matched controls). Unexpectedly, she had reduced sensitivity to pressure pain and vibration detection in the feet, which may relate to trophic changes in the skin following prolonged erythromelalgia.

### 3.2 Confirmation of the Na_v_*1.7* mutation

DNA sequence analysis revealed a heterozygous missense mutation in SCN9A gene, which encodes Na_v_1.7 (a C to T substitution at 2572), resulting in the substitution of leucine 858 with phenylalanine (L858F). This mutation has previously been described in a Canadian and Chinese family and is associated with a severe phenotype with an early onset of symptoms [13,18]. DNA sequence analysis of the parents was not performed, as neither was clinically affected, suggesting a de novo mutation. This mutation has previously been shown to cause a hyperpolarizing shift in channel activation [9,17] and an enhanced response to slow depolarizations; cooling can shift the activation midpoint of L858F in a depolarizing direction, bringing the threshold of activation of the mutant channel closer to wild-type Na_v_1.7 [17]; this provides a biophysical correlate of the relief of pain by cooling in patients with this mutation.

### 3.3 Psychophysics

**Fig. 4:**
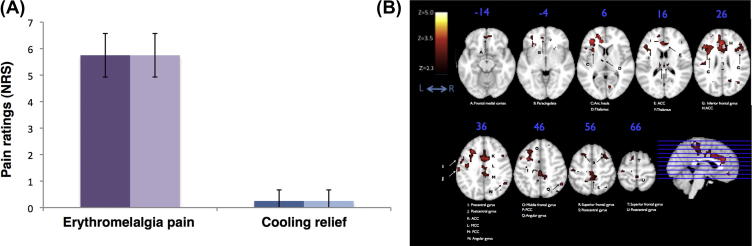
(A) Psychophysical data. Verbally reported pain intensity (solid) and unpleasantness (opaque) ratings averaged over the 5-minute blocks of erythromelalgia-associated heat pain (purple) and blocks of pain relief (blue) across both sessions. Error bars represent SD from the mean. (B) Brain perfusion data from the erythromelalgia case study acquired with the multi-TI whole brain pseudo-continuous arterial spin labelling sequence. Averaged zstat maps (n = 1) of perfusion activation superimposed on the MNI152 standard template brain (fixed effects, cluster corrected; *z*: 2.3–5.0, *P* < 0.05). Perfusion maps represent the contrast of the subject’s erythromelalgia-associated pain vs blocks of cooling pain relief. The sagittal slice shows the column of activation across the whole brain. Axial slices (one slice every 10 mm in ascending order) correspond to the plane indicated in the sagittal slice.

Fig. 4A shows the mean pain intensity ratings for the baseline warming and cooling stimuli. The mean pain intensity rating for each condition was (warming = 5.75, SE = 0.821; cooling = 0.25, SE = 0.41). The mean pain unpleasantness rating during warming was 5.75, SE = 0.41. During phases of cooling, the patient always rated the pain intensity and unpleasantness as <1 (or, not painful). There was a significant difference in pain intensity (Student *t*-test, *P* < 0.001) and unpleasantness (Student *t*-test, *p* < 0.001) across the conditions. To note: the subject self-reported that her left foot was always in more pain on each scan session. The subject consistently rated every cooling block as very pleasant (8 out of 10).

### 3.4 Perfusion imaging data: pain contrast

Significant changes in CBF generated by the contrast of Baseline pain > Cooling relief are shown in Fig. 4B. Coloured regions represent voxels with suprathreshold *t*-statistics generated from the FEAT analysis of variance across sessions. Significant increases in CBF occurred in many regions shown previously to be involved in processing acute [2], phasic [31], and tonic [19,29,30] pain. Active regions include: bilateral thalamus, primary somatosensory cortex (S1), inferior frontal gyrus, anterior, mid and posterior cingulate, right caudate, right putamen, and the left anterior insula. No significant changes in CBF were observed in the reverse contrast of Cooling relief > Baseline pain. All coordinates are in Montreal Neurological Institute space.

## 4 Discussion

In this study we have used a composite diagnostic experimental approach incorporating QST, genetic sequencing, and perfusion imaging to study a patient presenting with pain and erythema of the extremities. Genetic testing showed that these symptoms were due to the painful channelopathy IEM (due to a L858F Na_v_1.7 mutation). QST demonstrated hypersensitivity to heat and confirmed the temperature dependence of her tonic pain. Finally, a novel perfusion imaging approach was used to investigate the neural correlates of the patient’s pain vs phases of cooling relief.

This patient has a severe form of IEM; she was noted to have erythematous feet at birth and although onset of this disorder has previously been described as early as 1–2 years of age, we have not seen any previous reports of congenital onset [14]. She has always complained of bilateral foot pain exacerbated by warmth and relieved by cooling. Initially her symptoms were episodic, however, from her second decade she developed constant foot pain at room temperature that could only be relieved by cooling. IEM occurs as a consequence of gain of function mutations in the voltage-gated sodium channel Na_v_1.7 [13,14,20]. The patient described here was found to have a substitution of the amino acid phenylalanine for leucine at codon 858 of Na_v_1.7. This is within the S4/5 linker of domain 2 of the channel. This mutation has previously been described and is associated with a severe erythromelalgia phenotype with a young age of onset in a Canadian [13] and a Chinese kindred [18].

Na_v_1.7 mutations causing IEM are highly penetrant and give rise to increased dorsal root ganglion cell hyperexcitability (and hence pain) via a number of alterations in channel function, including: a hyperpolarizing shift in channel activation, slowed deactivation, and an enhanced response to slow ramp-like stimuli (ramp currents) [14]. Particular mutations have distinct effects on channel function and there is a complex relationship between these changes and the clinical severity: the greater the hyperpolarizing shift in activation, the earlier the age of onset [20]. However, mutations that also enhance slow inactivation reduce channel availability and lead to a milder phenotype [17]. The L858F mutation present in our patient has been reported to cause a large hyperpolarizing shift in voltage-dependent activation with no effect on slow inactivation. These biophysical changes therefore correlate with the severe phenotype [9,16]. Interestingly, cooling has been shown to shift the activation midpoint of L858F in a depolarizing direction, bringing the threshold of activation of the mutant channel closer to wild-type Na_v_1.7 [17]. The patient reported here demonstrated noxious heat hypersensitivity on QST of the feet, and the level of her tonic pain was strongly modulated by ambient temperature (being rapidly relieved by cooling). We exploited this phenomenon to investigate the imaging correlates of her tonic pain.

The patient showed robust activation of key pain sensory and pain-affect regions during phases of IEM-related pain that ceased during cooling relief. Pain was related to increased perfusion in the bilateral thalamus, S1, inferior frontal gyrus, anterior, mid and posterior cingulate, right putamen, right caudate, and the left anterior insula. There was a lateral dominance of activity in several regions that was contralateral to her greatest foot pain, as would be expected. Our data align with previous tonic pain studies using ASL and positron emission tomography. For example, regions such as the insula, thalamus, putamen, cingulate, and S1 have all been shown previously to play a role in ongoing pain states. While the present study is unable to interrogate the mechanisms of tonic pain processing, these data provide exciting additional evidence that many regions activated in well-known BOLD fMRI studies of acute pain are also found to be active during a chronic pain state [20,21].

Because of the robust relieving action induced by cooling at the site of the erythromelalgia pain, it was possible to image the attenuation of brain regions constituent of a multidimensional pain network. Cooling relief caused a cessation of activation in these regions. No additional regions were observed to be more active during cooling compared to pain. While this could be due to low signal-to-noise related to the single-subject nature of the case, it is clear that the cooling stimulus acting at the site of the erythema is a major component driving pain relief. This would be consistent with the effects of cooling on mutant L858P Na_v_1.7 function and presumably correlates with reduced ongoing nociceptor activity following cooling. Microneurography has been performed in a cohort of patients with primary erythromelalgia, and this demonstrated altered C-fibre function, including enhanced activity-dependent slowing of afferent units and increased spontaneous activity [28]. The effects of cooling on C-fibre function were not investigated in this report.

Interestingly, pain relief in the form of cessation or reduction of a noxious stimulus can be thought of as a reward. Occasionally, this sensation is pleasurable. Previous work by Becerra et al. (2001, 2006) and Baliki et al. (2010) provide strong evidence that activity within the nucleus accumbens (NAc) tracks phases of pleasurable relief related to pain offset [3–5]. Similarly, because of the block design used in the current study, we predict that increased activation within the NAc during phases of cooling would be visible as the cooling blocks reflect a kind of pleasurable pain-offset triggered by reduced nociceptor function. While the present investigation was unable to observe significant perfusion changes from a whole-brain analysis during cooling relief; it is possible that subsequent investigations focused purely on the NAc may provide insight into the higher cognitive processes underlying the experience of pleasure related to cooling-induced relief of erythromelalgia-associated pain.

The present study confirms the temperature dependence of the L858F Na_v_1.7 mutation and further demonstrates the related effects on pain processing in the cortex by using a pCASL fMRI approach. Recent advances in ASL pulse sequence programming have improved the technique such that pCASL benefits from greatly enhanced signal-to-noise ratio and reproducibility compared to other commonly used ASL approaches [8,15]. However, it is possible that the technique is still ill suited to do single-subject investigations without the capacity to do multiple repeat scans within the subject, as we have done here. Follow-up studies may further benefit by defining a priori hypotheses about specific brain regions of interest such that more region-of-interest-driven analyses may be used that do not necessitate whole-brain corrections.

A key goal of this work was to employ a composite investigative approach to study a chronic pain condition. From previous pain neuroimaging studies, it is well known that pain is a complex, subjective experience constituent of sensory, psychological, cognitive and neuropharmacological factors. This is made even more complex in the context of chronic pain, where unique combinations of structural and functional abnormalities could possibly be used to define different types of pain disease states [36]. The success of future human pain studies relies partly on the capacity to encapsulate this complexity. As a case study, the present work attempted to do this by employing a multivariate investigative approach that interrogated the pain from the level of ion channel dysfunction on peripheral nociceptors to higher cortical pain processing in the brain. Interpreting the brain imaging data, although in a single subject, it is clear that the optimised ASL approach used is capable of imaging the effect of chronic pain and relief across a whole brain. Additionally, our data provide evidence that in the case of erythromelalgia, it appears as though cooling relief produces analgesia via attenuation of peripheral inputs rather than via top-down brain mechanisms related to pleasant relief.

We hope that future studies employing a composite, “molecule-to-man” approach as we have done here will help develop a mechanism-based understanding of chronic pain in patients.

## Conflict of interest statement

The authors have no conflicts of interest to declare.

## Appendix A Supplementary data

Supplementary Table 1 Erythromelalgia pain versus cooling relief. Brain responses to Erythromelalgia-associated pain versus the cooling relief (BASE >COOL). Note: PCC, posterior cingulate cortex; M1, precentral gyrus; S1, postcentral gyrus; L, left; R, right.
