# Supplementary material for: Imaging the neural correlates of neuropathic pain and pleasurable relief associated with inherited erythromelalgia in a single subject with quantitative arterial spin labelling
Source: Pain. 2012 May;153(5):1122–7. doi: 10.1016/j.pain.2011.12.012 (PMC3438450; doi:10.1016/j.pain.2011.12.012)
Supplement: Supplementary Table 1 — Erythromelalgia pain versus cooling relief. Brain responses to Erythromelalgia-associated pain versus the cooling relief (BASE >COOL). Note: PCC, posterior cingulate cortex; M1, precentral gyrus; S1, postcentral gyrus; L, left; R, right. [file mmc1.doc]

| **cluster** | **zstat** | **MNI** | **Laterality** | **ROI** |
| --- | --- | --- | --- | --- |
| **27** | 3.4 | (61 80 36) | L | Frontal orbital cortex |
| **26** | 2.9 | (37 71 67) | R | Superior frontal gyrus |
| **25** | 2.89 | (27 31 51) | L | Lateral occipital cortex |
| **24** | 3.04 | (19 36 57) | R | Angular gyrus |
| **23** | 2.92 | (40 25 39) | R | Intracalcarine cortex |
| **22** | 3.05 | (65 37 51) | L | Angular gyrus |
| **21** | 3.1 | (40 90 46) | R | Superior frontal gyrus |
| **20** | 2.67 | (27 33 65) | L | Lateral occipital cortex |
| **19** | 2.63 | (26 78 35) | R | Frontal orbital cotex |
| **18** | 2.74 | (34 74 33) | R | Putamen |
| **17** | 2.74 | (36 81 60) | R | Frontal pole |
| **16** | 2.51 | (39 53 44) | R | Thalamus |
| **15** | 2.39 | (32 83 41) | R | White Mater |
| **14** | 2.38 | (39 71 38) | R | Caudate |
| **13** | 2.35 | (54 41 51) | R | PCC |
| **12** | 2.49 | (35 22 40) | R | White Mater |
| **11** | 2.56 | (50 67 36) | L | Caudate |
| **10** | 2.3 | (56 32 49) | L | Precuneus |
| **9** | 2.45 | (54 41 51) | L | white matter |
| **8** | 2.4 | (30 27 38) | R | white matter |
| **7** | 2.15 | (64 5763) | L | M1/S1 |
| **6** | 2.38 | (21 40 62) | R | Supramarginal gyrus |
| **5** | 2.14 | (40 88 54) | R | Frontal pole |
| **4** | 2.04 | (16 41 58) | R | Supramarginal gyrus |
| **3** | 2.36 | (54 91 50) | L | Frontal pole |
| **2** | 2.14 | (52 45 43) | L | white matter |
| **1** | 2.08 | (43 58 37) | R | Thalamus |

**Supplementary Table 1**: Erythromelalgia pain versus cooling relief. Brain responses to Erythromelalgia-associated pain versus the cooling relief (BASE >COOL). Note: PCC, posterior cingulate cortex; M1, precentral gyrus; S1, postcentral gyrus; L, left; R, right.
